# The long noncoding RNA NARL regulates immune responses *via* microRNA-mediated NOD1 downregulation in teleost fish

**DOI:** 10.1016/j.jbc.2021.100414

**Published:** 2021-02-11

**Authors:** Weiwei Zheng, Qing Chu, Tianjun Xu

**Affiliations:** 1 Laboratory of Fish Molecular Immunology, College of Fisheries and Life Science, Shanghai Ocean University, Shanghai, China; 2 Laboratory of Marine Biology and Biotechnology, Qingdao National Laboratory for Marine Science and Technology, Qingdao, China; 3 Key Laboratory of Exploration and Utilization of Aquatic Genetic Resources, Shanghai Ocean University, Ministry of Education, Shanghai, China; 4 National Pathogen Collection Center for Aquatic Animals, Shanghai Ocean University, Shanghai, China

**Keywords:** long noncoding RNA, microRNA, ceRNA, NOD1, immune responses, ceRNA, competing endogenous RNA, EPC, *Epithelioma papulosum cyprinid*, lncRNA, long noncoding RNA, LPS, lipopolysaccharides, MAPK, mitogen-activated protein kinase, MDP, muramyl dipeptide, MIC, *M. miiuy* intestine cell, NARL, NOD1 antibacterial and antiviral-related lncRNA, NF-κB, nuclear factor-κB, NLR, nucleotide-binding oligomerization domain-like receptor, NOD1, nucleotide oligomerization domain 1, PRR, pattern recognition receptor, RIP, RNA immunoprecipitation, SCRV, *Siniperca chuatsi rhabdovirus*

## Abstract

Increasing evidence shows that the long noncoding RNA (lncRNA) is a major regulator and participates in the regulation of various physiological and pathological processes, such as cell proliferation, differentiation, metastasis, and apoptosis. Unlike mammals, however, the study of lncRNA in lower invertebrates is just beginning and the extent of lncRNA-mediate regulation remains unclear. Here, we for the first time identify an lncRNA, termed nucleotide oligomerization domain 1 (NOD1) antibacterial and antiviral-related lncRNA (NARL), as a key regulator for innate immunity in teleost fish. We found that NOD1 plays an important role in the antibacterial and antiviral process in fish and that the microRNA miR-217-5p inhibits NOD1 expression and thus weakens the NF-κB and the IRF3-driven signaling pathway. Furthermore, our results indicated that NARL functions as a competing endogenous RNA (ceRNA) for miR-217-5p to regulate protein abundance of NOD1; thus, invading microorganisms are eliminated and immune responses are promoted. Our study also demonstrates the regulation mechanism that lncRNA NARL can competitive adsorption miR-217-5p to regulate the miR-217-5p/NOD1 axis is widespread in teleost fish. Taken together, our results reveal that NARL in fish is a critical positive regulator of innate immune responses to viral and bacterial infection by suppressing a feedback to NOD1-NF-κB/IRF3-mediated signaling.

Innate immunity is the first line of host defense and mainly composes of pattern recognition receptors (PRRs) and their signal pathways. Nucleotide-binding oligomerization domain-like receptors (NLRs) are part of the cytosolic PRRs and play key roles in response to various bacterial infections (1). As the members of the NLR family, nucleotide oligomerization domain (NOD) 1 and NOD2 are important for epithelial and myeloid cell signaling in response to cytosolic bacterial peptidoglycans, muramyl dipeptide (MDP) and G-D-glutamyl-mesodiaminopimelic acid (iE-DAP) (2, 3). NOD1 can monitor bacterial intrusions in cells and promotes the activation of the nuclear factor-κB (NF-κB) and mitogen-activated protein kinase (MAPK) signaling pathways through RIP2 kinase (4, 5). In lower vertebrates, increasing pieces of evidence show that NOD1 is widespread in fish and participates in the regulation of antibacterial immune response after pathogen infection (6, 7, 8, 9, 10). Recently, researchers have demonstrated that fish NOD1 plays an important role in recognizing lipopolysaccharides (LPS) and iE-DAP, as well as initiating downstream antiviral signaling pathways by identifying RNA virus (9, 10, 11). Unlike mammals, TLR4, as the central protein that recognizes LPS, does not exist in most fish. Therefore, it was found that fish NOD1 can recognize LPS and iE-DAP and eventually causes an inflammatory response that could elucidate the resistance of fish against bacterial infections.

Recently, a series of regulatory factors involved in the regulation of the NOD1 signaling pathway has been found. CENTB1 and E3 Ligase RNF34 regulate the NOD1 signaling pathway through directly regulating the expression of NOD1 (12, 13); the molecular chaperone HSP90 regulates the NOD1 signal pathway by regulating the stability of NOD1 (14); it was also found that SGT1 can act as a positive regulator of the NOD1 signaling pathway (15). In addition to protein regulatory factors, microRNA (miRNA) has also been found to play an important role in the NOD1 signaling pathway in recent years. For example, PPARγ-regulated miR-125a could target NOD1 and regulate NOD1-mediated angiogenesis (16). Since NOD1 is an important intracellular receptor that recognizes LPS in fish, the elucidation of the regulatory mechanism of the NOD1 signaling pathway in fish is even more important. Our laboratory had found for the first time that miR-144 and miR-217-5p can target and negatively regulate the expression of NOD1 to attenuate the NOD1-mediated inflammatory response in fish (17).

The long noncoding RNA (lncRNA) is defined as RNA with noncoding potential and larger than 200 nucleotides length (18, 19). An increasing number of studies have shown that lncRNA is a major regulator and participates in the regulation of cell proliferation, differentiation, metastasis, and apoptosis (20, 21). Recent studies also show that lncRNAs can play the role of competitive endogenous RNAs (ceRNA) by adsorbing miRNA, thus regulating the binding of miRNA and mRNA. Until now, the function of lncRNAs has only been widely studied in mouse and human, such as lncRNA LINC01133, MIAT, or UCA1, all can be used as ceRNA to competitively bind miRNA to regulate mRNA (22, 23, 24). However, the function mechanism of lncRNA in lower vertebrates is still poorly understood.

Teleost is a representative population of lower vertebrates and an important part of early vertebrate evolution, so it is considered an excellent biological model in immunology research. The teleost fish not only has a very important significance in biological evolution and immunity but also its status in the aquatic industry is not to be underestimated. However, artificially cultivated fishes are extremely vulnerable to various pathogens, especially Gram-negative bacteria and RNA virus, such as *Aeromonas hydrophila*, *Edwardsiella tarda*, *Vibrio harveyi*, and *Siniperca chuatsi rhabdovirus* (SCRV) (25, 26). The mechanism that induces fish disease and how to prevent and solve these fish diseases are problems that need to be discovered and solved urgently. LPS and iE-DAP are pathogenic components carried by various Gram-negative bacteria and can induce an inflammatory response in the host (27, 28). At present, the pathogenic mechanism of Gram-negative bacteria and RNA virus has been relatively comprehensive in mammals. However, the research process of teleost fish is different from that of mammals and has great limitations and shortcomings as the slow development of research technology and difficulty in obtaining materials.

In this study, we firstly identified a ceRNA regulatory loop involved in innate immune responses in teleost fish. Our previous work has revealed that miR-217-5p can negatively regulate NOD1 and suppress the antibacterial immune response in miiuy croaker (*Miichthys miiuy*) (17). Herein, we further suggest that miR-217-5p can negatively regulate NOD1 and can suppress the antiviral response in miiuy croaker. Furthermore, we found that an lncRNA, named NARL, can act as a ceRNA for miR-217-5p to facilitate NOD1 expression, thus modulating innate immune responses. Our research not only contributes to the understanding of the ceRNA network mechanism in teleost fish but also provides a reference for the importance of lncRNA in the innate immune response in lower vertebrates.

## Results

### Characterization of NARL

**Figure 1:**
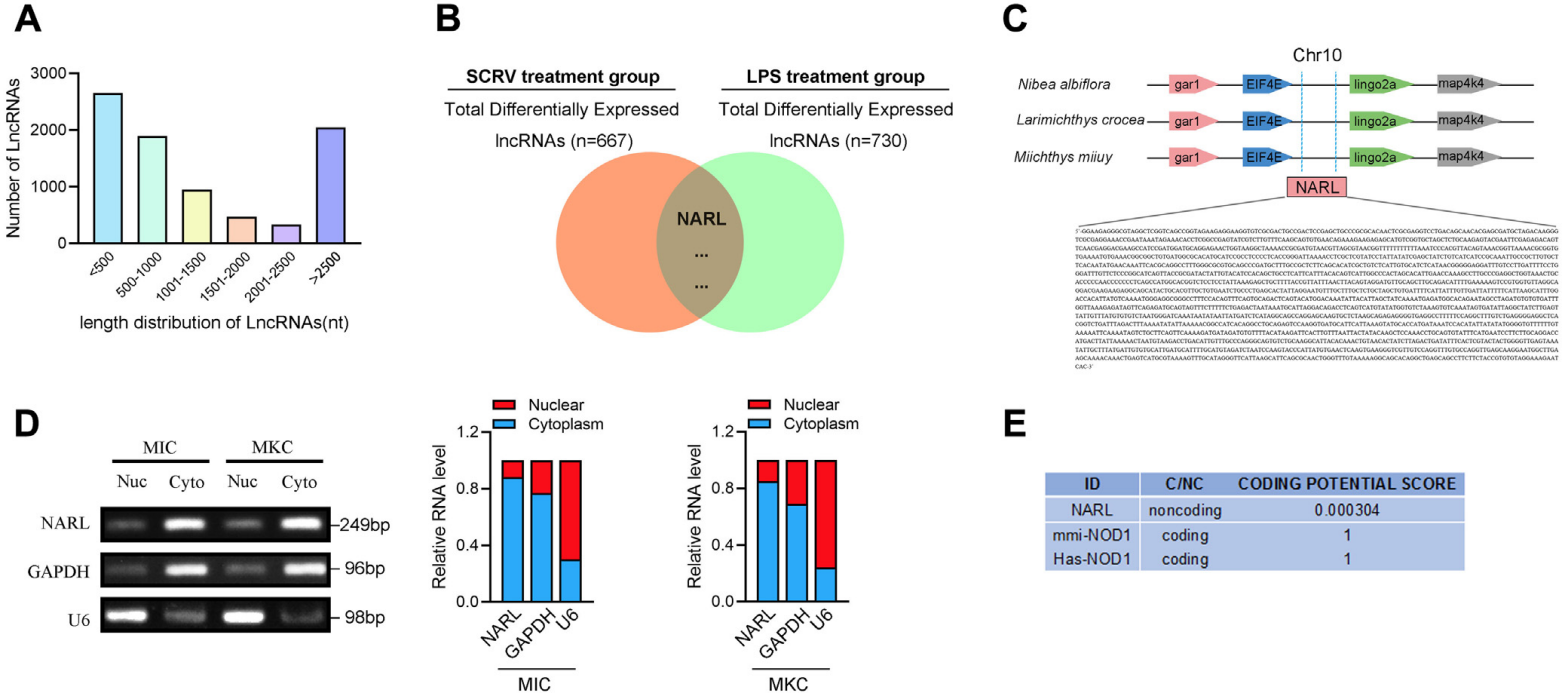
**Characterization of NARL.***A*, the number of diverse length distributions of lncRNAs. *B*, Venn diagram of the differentially expressed lncRNAs upon LPS or SCRV treatment. A cluster heat map presented the significantly dysregulated lncRNAs in LPS-treated spleen tissues compared with untreated tissues. The red and blue stripes represented the high and low expression, respectively. *C*, schematic of the NARL locus. NARL locates on miiuy croaker chromosome 10. *D*, NARL was mainly localized in the cytoplasm. RNA isolated from nuclear (Nuc) and cytoplasm (Cyto) was used to analyze the expression of NARL by RT-PCR; *n* = 3. *E*, NARL was predicted to be noncoding RNA. The RNA sequences of NARL were put into the Coding Potential Calculator (CPC) program, which was predicted to be noncoding RNAs. *hsa*-NOD1, *Homo sapiens* NOD1 gene; LPS, lipopolysaccharides; *mmi*-NOD1, *M. miiuy* NOD1 gene.

Increasing pieces of studies have shown that lncRNA can play a regulatory role as a spongy body adsorbing miRNA and interfere with the host’s immune signaling pathways through the sponge mechanism (29). We treated miiuy croaker with LPS and SCRV to identify whereas lncRNAs are potentially involved in the regulation of infection, and then the expression of lncRNAs in the spleen tissues of the treated group and the untreated group was analyzed by RNA-seq data. In total, 8942 distinct lncRNA candidates were found. The number of diverse length distribution of lncRNAs was different, and the length of most lncRNAs was less than 500 nucleotides (Fig. 1*A*). As shown in Figure 1*B*, we found that a highly expressed lncRNA (named NARL according to the participating function) is copresent in the differentially expressed genes obtained after LPS stimulation and SCRV infection (Table S2).

To characterize the complete sequence of NARL, the single-molecule full-length transcript sequencing was used and demonstrated that the length of NARL was 1992 base pairs (bp) and NRAL locates on miiuy croaker, *N. diacanthus*, and *L. crocea* chromosome 10, between gene *EIF4E* and gene *lingo2a*, and consists of only one exon (Fig. 1*C*). Then, we validated that NARL was mainly distributed in the cytoplasm by cytoplasm/nucleus fraction assay both in MIC cells and in MKC cells (Fig. 1*D*). Consistent with NARL being an ncRNA, no putative protein is conserved in all species, and the CPC (coding potential calculator) computational algorithm predicts that NARL has a very low coding potential (Fig. 1*E*).

### NARL enhances the host innate immunity

**Figure 2:**
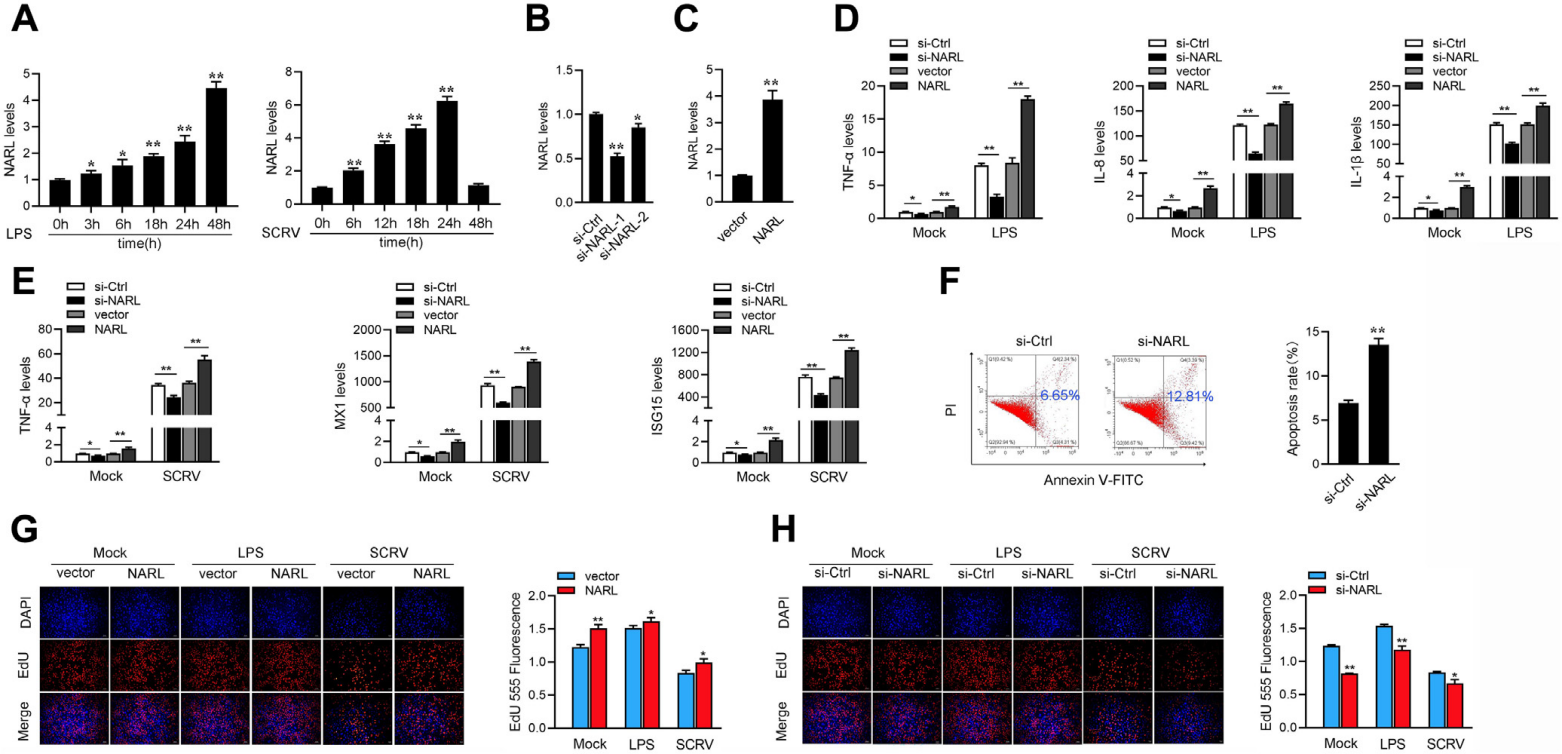
**NARL enhances host innate immunity.***A*, LPS or SCRV induces an increase of NARL expression. The expression levels of NARL in MIC cells were measured by qPCR at indicated time after LPS or SCRV stimulation. *B*, the effect of si-NARL-1 and si-NARL-2 on endogenous NARL expression. MIC cells were transfected with two NARL-specific siRNAs (si-NARL-1 and si-NARL-2) or si-Ctrl (*B*) with for 48 h, then NARL expression was determined by qPCR. *C*, MIC cells were transfected with NARL-specific siRNAs (si-NARL-1) or si-Ctrl, and pcDNA3.1 vector and NARL expression plasmid for 48 h. And the cells were treated with LPS for 6 h. The expressions of TNF-α, IL-8, and IL-1β were analyzed by qPCR. *D*, the effect of NARL expression plasmid on endogenous NARL expression. MIC cells were transfected with pcDNA3.1 vector or NARL expression plasmid with for 48 h, then NARL expression was determined by qPCR. *E*, MIC cells were transfected with NARL-specific siRNAs (si-NARL-1) or si-Ctrl, and pcDNA3.1 vector and NARL expression plasmid. At 24 h posttransfection, MIC cells were treated with SCRV for 24 h. The expressions of TNF-α, MX1, and ISG15 were analyzed by qPCR. *F*, the effect of NARL knockdown on cell apoptosis was analyzed by flow cytometric cell apoptosis assays. *G* and *H*, cell proliferation was assessed by EdU assays in MIC cells transfected with pcDNA3.1 vector or NARL expression plasmid and with si-circ or NC (*G*) and si-NARL or si-Ctrl. (*H*) Effect of NARL on cell viability after LPS or SCRV stimulation. MIC cells were transfected with si-Ctrl, si-NARL, pcDNA3.1 vector, or NARL for 48 h, then treated with LPS or SCRV. Cell viability assay was measured. All data represented the mean ± SD from three independent triplicated experiments. ∗*p* < 0.05. LPS, lipopolysaccharides.

We first detected the NARL expression under different LPS or SCRV stimulation times to verify the results of RNA-seq. As shown in Figure 2*A*, we found that the expression of NARL was upregulated after LPS and SCRV stimulation. Two small interfering RNAs against NARL (si-NARL-1 and si-NARL-2) and the overexpression plasmid of NARL were constructed to explore the biological functions of NARL. As expected, both two siRNAs can inhibit the expression of NARL, and si-NARL-1 could induce higher inhibitory efficiency (Fig. 2*B*), so we chose si-NARL-1 to continue the follow-up experiment. And the NARL overexpression plasmid could significantly increase NARL expression levels (Fig. 2*D*). Inflammatory cytokines and IFN-stimulated genes (ISGs) play an important role in the innate immune response; thus, we focused on investigating the function of NARL in regulating the expression of inflammatory cytokines and ISGs genes. As shown in Figure 2*C*, knockdown or overexpression NARL can significantly inhibit or promote the expression levels of TNF-α, IL-8, and IL-1β upon LPS stimulation. Then, knockdown or overexpression NARL also can significantly inhibit or promote the expression levels of TNF-α, MX1, and ISG15 upon SCRV infection (Fig. 2*E*). Furthermore, cell apoptosis analysis showed that NARL knockdown significantly increased the proportion of apoptotic cells (Fig. 2*F*). We conducted EdU assays and ATP activity assay to examine cell proliferation and cell viability for further explore the role of NARL in innate immunity. As shown in Figure 2, *G* and *H*, the results showed that knockdown of NARL considerably decreased the percentages of EdU-positive cells and cell viability, whereas it greatly increased when NARL was overexpressed. Previous studies have indicated that the low concentration of LPS can stimulate cell proliferative activity (30). These results suggested that NARL can promote the proliferation and viability of miiuy croaker cell lines. To summarize, the data suggest that NARL plays as a positive modulator in regulating inflammatory responses, as well as cell proliferation and cell viability. The changes in cell proliferation and cell apoptosis are a result of the regulation effect of NARL on innate immune responses. In other words, NARL can positively regulate the antibacterial responses or antiviral response and upregulate the expression of inflammatory cytokines or antiviral genes, reducing the attack of pathogen to cells, promoting cell proliferation, and reducing cell apoptosis.

### NARL can regulate miR-217-5p expression and activity

**Figure 3:**
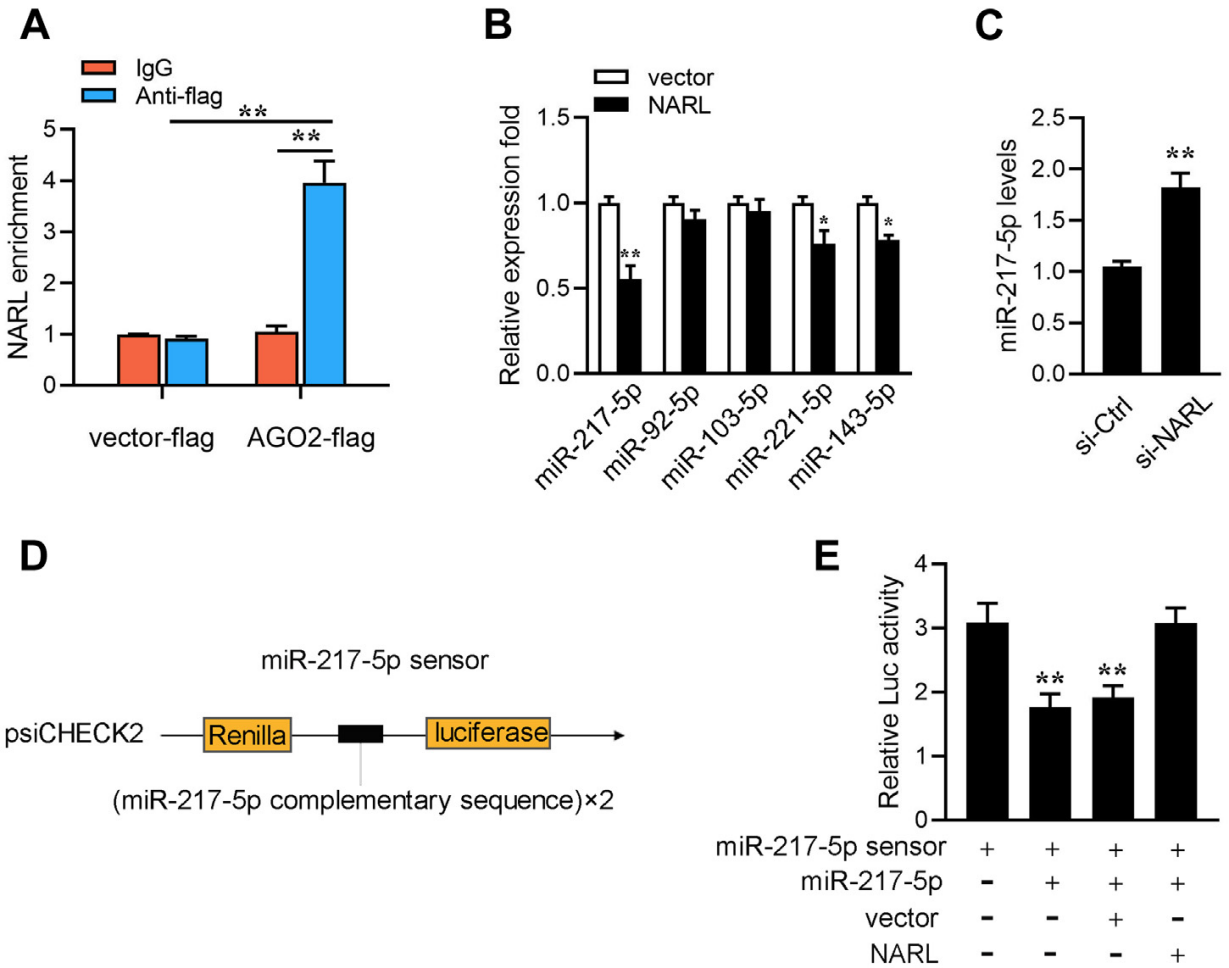
**NARL regulates miR-217-5p expression and activity.***A*, the Ago-RIP assay for the amount of NARL in MIC cells transfected Ago2-flag or pcDNA3.1-flag. *B*, a schematic drawing showing the putative binding sites of the miRNAs associated with NARL. *C*, knockdown of NARL upregulated miR-217-5p expression. MIC cells were transfected with si-NARL or si-Ctrl for 48 h. The expression of miR-217-5p was measured by qPCR. *D*, miR-217-5p sensor construct. The miR-217-5p sensor was constructed by inserting two copies of perfectly matched miR-217-5p fragments into the psiCHECK-2 vector. *E*, NARL reduces miR-217-5p activity. MIC cells were transfected with mimics, control vector, or NARL overexpression plasmid, together with miR-217-5p sensor. The luciferase activity was analyzed and normalized to renilla luciferase activity. All data represented the mean ± SD from three independent triplicated experiments. ∗∗*p* < 0.01.

Many cytoplasmic lncRNAs have been reported to act as ceRNAs by competitively binding miRNAs (29, 31). Through the cytoplasm/nucleus fraction assay experiments, we have been able to determine that NARL is mainly distributed in the cytoplasm. Therefore, to further confirm whether NARL acts as a ceRNA, we first inserted the Argonaute-2 (Ago2) sequence into the pcDNA3.1-flag vector and obtained the Ago2-flag expression plasmid. We next construct the RIP assay with Ago2-flag in MIC cells. As shown in Figure 3*A*, NARL is significantly enriched in Ago2-containing micro-ribonucleoprotein complexes, which suggests that the miRNAs can directly bind to NARL. Then, we used Targetscan and miRanda softwares to predict miRNAs that can potentially target NARL and that the five microRNAs (including miR-217-5p, miR-103-5p, miR-143-5p, miR-221-5p, and miR-92-5p) were predicted to have a high probability of combining to NARL. Then, we used qRT-PCR to detect these five miRNAs’ expressions in MIC cells when overexpressing NARL expression plasmid, and the result shows that the expression of miR-217-5p is significantly downregulated compared with the other four miRNAs (Fig. 3*B*). Meanwhile, we found that the expression of miR-217-5p significantly increased when the NARL was silenced (Fig. 3*C*). To test whether NARL can affect miR-217-5p activity, we constructed miR-217-5p sensor. The miR-217-5p sensor was constructed by inserting two copies of perfectly matched miR-217-5p fragments into the psiCHECK-2 vector (Fig. 3*D*). Then, we transfected with the miR-217-5p sensor into cells, along with miR-217-5p, control vector, or NARL overexpression plasmid, suggesting that NARL specifically sponged miR-217-5p, thereby preventing it from inhibiting luciferase activity (Fig. 3*E*). Taken together, these data suggest that NARL could regulate miR-217-5p expression and activity, and NARL may function as a sponge of miR-217-5p.

### NARL is able to directly bind miR-217-5p

**Figure 4:**
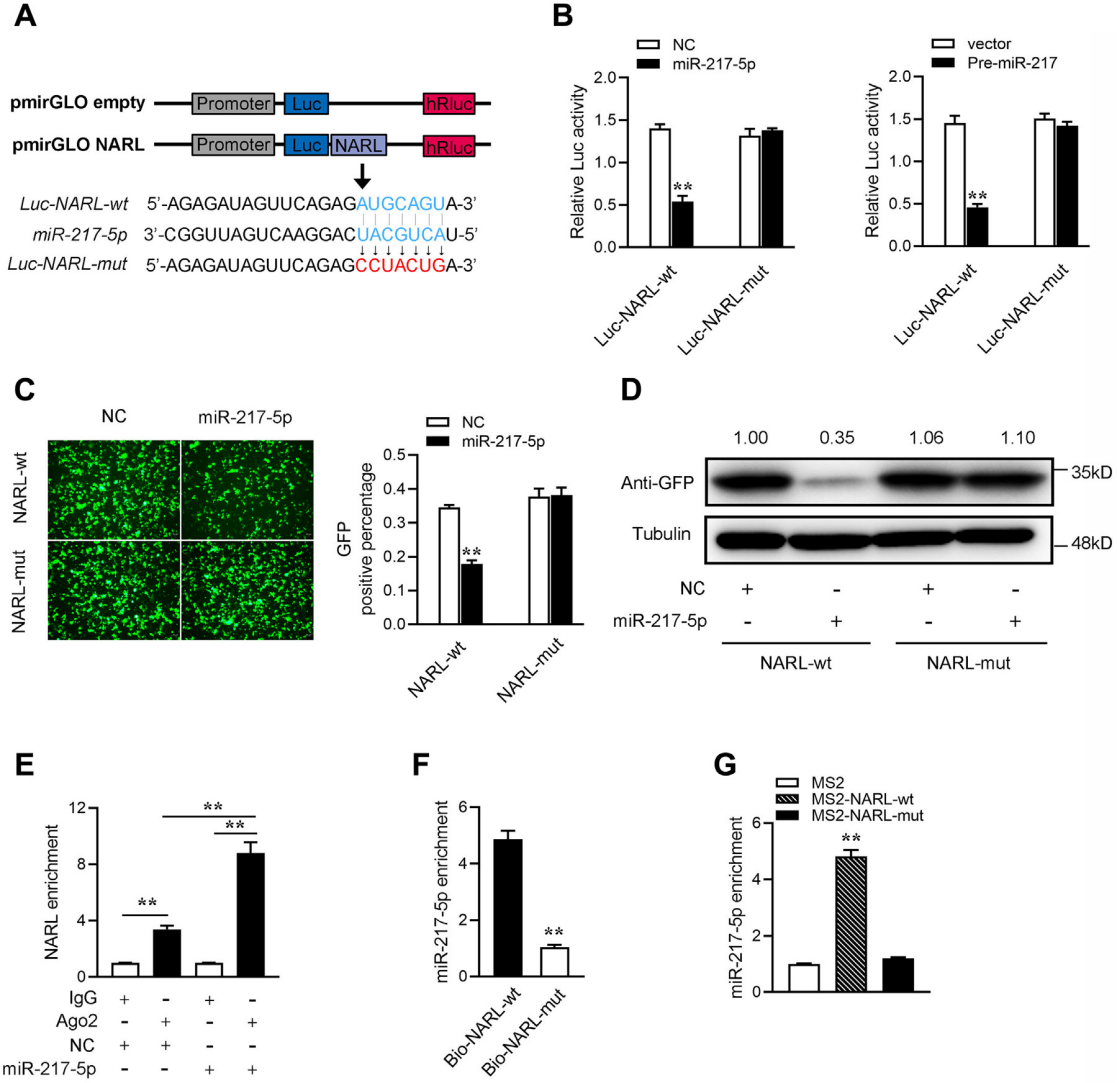
**NARL is able to directly bind miR-217-5p.***A*, schematic illustration of NARL-wt and NARL-mut luciferase reporter vectors. *B*, the relative luciferase activities were detected in EPC cells after cotransfection with NARL-wt or NARL-mut, miR-217-5p or NC, and Pre-miR-217 or pcDNA3.1 vector. *C* and *D*, NARL downregulated GFP expression. EPC cells were cotransfected with NARL-wt or NARL-mut and miR-217-5p or NC. The fluorescence intensity (*C*) and the GFP expression (*D*) were evaluated by enzyme-labeled instrument and western blotting, respectively. *E*, the Ago-RIP assay was executed in MIC cells after transfection with miR-217-5p and NC, followed by qPCR to detect NARL expression levels. *F*, RNA pull-down assay was executed in MIC cells, followed by qPCR to detect the enrichment of NARL and miR-217-5p. *G*, the MS2-RIP assay was executed in MIC cells after transfection with pcDNA3.1-MS2, pcDNA3.1-MS2-NARL, pcDNA3.1-MS2-NARL-mut, followed by PCR to detect the enrichment of miR-217-5p. All data represented the mean ± SD from three independent triplicated experiments. ∗*p* < 0.05; ∗∗*p* < 0.01.

We analyzed the sequence of NARL and found a binding site for miR-217-5p to investigate whether NARL can directly interact with miR-217-5p. Then, we constructed the NARL luciferase plasmid and the mutant plasmid with the mutated miR-217-5p binding site (Fig. 4*A*). The luciferase assay shows that miR-217-5p mimics and pre-miR-217 plasmid can both significantly inhibit the wild-type NARL luciferase plasmid activity, but have no effect on the mutated form (Fig. 4*B*). In addition, we inserted the wild-type or the mutant-type fragment of NARL into the mVenus-C1 vector and cotransfected with miR-217-5p to confirm that whether miR-217-5p can inhibit the levels of GFP. As shown in Figure 4*C*, miR-217-5p can significantly inhibit the levels of GFP, but cannot inhibit the level of the mutant type of NARL-GFP. To extend the findings, the western blotting assay has been conducted to examine the expression level of GFP protein (Fig. 4*D*). These results indicate that NARL may interact with miR-217-5p through the predicted miR-217-5p bind site.

Through the previous RIP experiment, we have known that NARL could directly bind to miRNAs (Fig. 3*A*). We thus further tested the ability of NARL to bind to miR-217-5p. To this end, the Ago2 RIP assays are performed in MIC cells by cotransfecting Ago2-flag and miR-217-5p. The results from qRT-PCR analysis suggested that NARL and miR-217-5p were efficiently pulled down by Ago2-flag (Fig. 4*E*). For further confirming the direct interaction between NARL and miR-217-5p, we performed biotin-avidin pull-down experiments to examine whether NARL can be pulled down by MIC cells that were transfected with biotinylated NARL or a mutated form, then harvested for pull-down assay. The result showed that miR-217-5p could pull down by biotinylated wild-type NARL (Fig. 4*F*). Then we constructed plasmids that recognize lncRNAs by MS2 protein. We insert the MS2-12X fragment into the pcDNA3.1, pcDNA3.1-NARL, and the mutated type of NARL plasmids (pcDNA3.1-NARL-mut). Additionally, we constructed a fusion expression plasmid of GFP and MS2 to produce MS2-GFP fusion protein (pcDNA3.1-MS2-GFP), and the protein can bind to MS2-12X and also GFP antibody. Therefore, if miRNAs can bind to NARL, miR-217-5p can be pulled down by the GFP-MS2-12X complex. Analysis of qPCR results showed that the miR-217-5p enrichment of NARL was significantly higher than that of mutant NARL and empty plasmid (Fig. 4*G*). In summary, these results indicated that NARL can directly bind to miR-217-5p, and NARL may act as a sponge of miR-217-5p.

### miR-217-5p suppresses innate immunity by targeting NOD1

**Figure 5:**
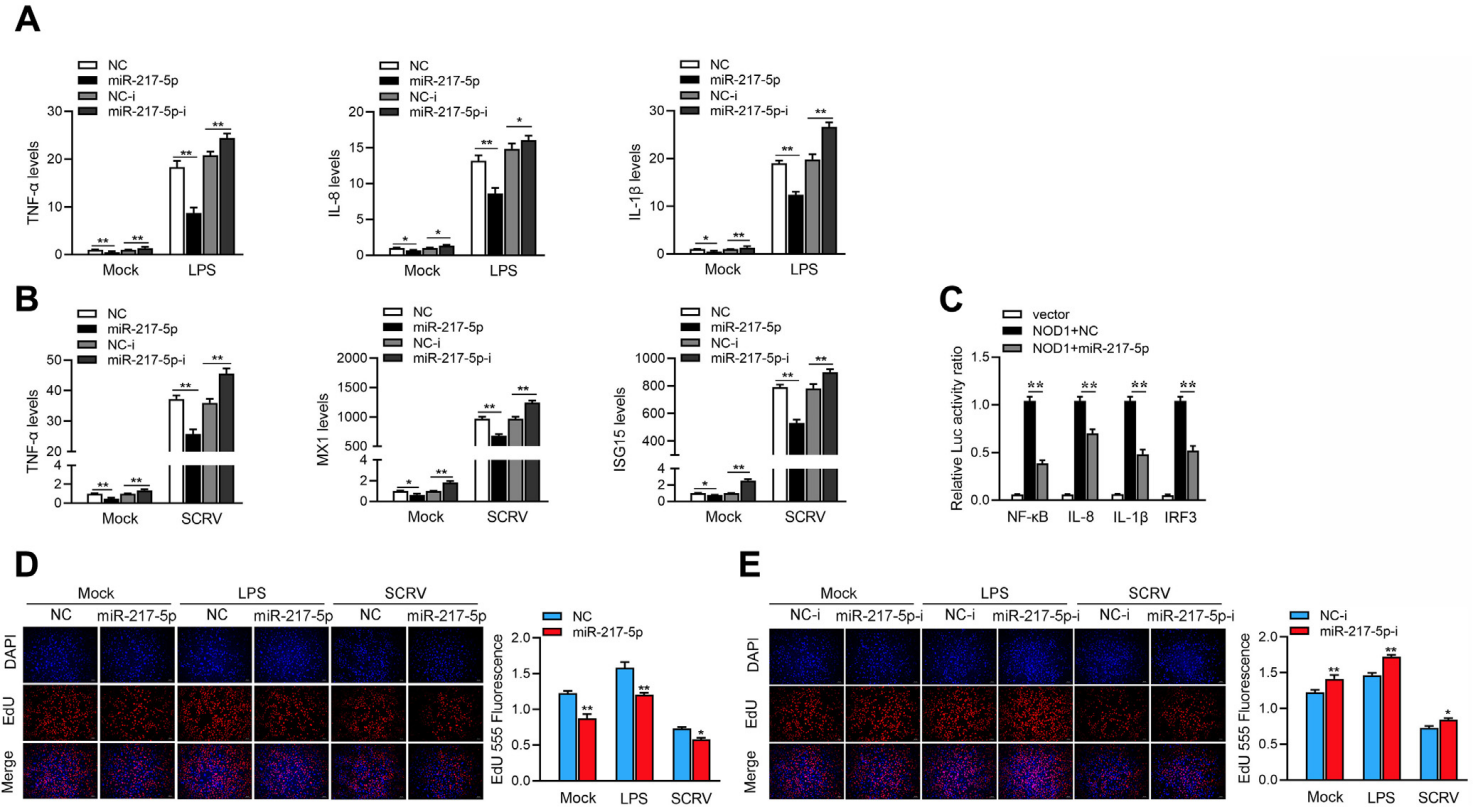
**miR-217-5p suppresses innate immunity by targeting NOD1.***A*, MIC cells were transfected with NC or miR-217-5p and NC-i or miR-217-5p-i for 48 h. The cells were treated with LPS for 6 h. The expressions of TNF-α, IL-8, and IL-1β were analyzed by qPCR. *B*, MIC cells were transfected with NC or miR-217-5p and NC-i or miR-217-5p-i. At 24 h posttransfection, MIC cells were treated with SCRV for 24 h. The expressions of TNF-α, MX1, and ISG15 were analyzed by qPCR. *C*, miR-217-5p could suppress NF-κB, IL-8, and IL-1β signaling. MIC cells were transfected with NC or miR-217-5p, together with NOD1 expression plasmid, phRL-TK Renilla luciferase plasmid, and luciferase reporter genes. At 48 h posttransaction, the luciferase activity was measured and normalized to renilla luciferase activity. Finally, we present the ratio of each group with NOD1 and NC cotransfection groups. *D* and *E*, cell proliferation was assessed by EdU assays in MIC cells transfected with NC or miR-217-5p (*E*) and with NC-i or miR-217-5p-i. All data represented the mean ± SD from three independent triplicated experiments. ∗*p* < 0.05. LPS, lipopolysaccharides.

To explore the role of miR-217-5p against the innate immune response, miR-217-5p and miR-217-5p inhibitors were transfected into MIC cells and stimulated by LPS, respectively. The results showed that certain inflammatory cytokines, including TNF-α, IL-8, and IL-1β, are significantly decreased or increased by the introduction of miR-217-5p mimics or miR-217-5p inhibitor upon LPS stimulation. Furthermore, we found that miR-217-5p or miR-217-5p inhibitor also could significantly decrease or elevated ISGs genes respectively, including MX1 and ISG15 (Fig. 5*B*). Here, we find that the effect of the miR-217-5p inhibitor on the expression of some of the innate immune response genes is not robust, but there are significant differences; we think that this may be related to the inhibitory efficiency of miRNA inhibitor, and background level of miR-217-5p is not high in cells. Then, NOD1-3’UTR plasmid, miR-217-5p, and reporter genes were cotransfected into EPC cells for further verifying that NOD1 can be regulated by miR-217-5p. The results showed that miR-217-5p could inhibit the NF-κB, IL-8, IL-1β, and IRF3 report genes’ luciferase activity by inhibiting NOD1 (Fig. 5*C*). Furthermore, we tested the effects of miR-217-5p and miR-217-5p-i on MIC cell proliferation, the results showed that miR-217-5p inhibited cell proliferation and miR-217-5p-i promoted cell proliferation under LPS or SCRV stimulation (Fig. 5, *D* and *E*). Combined with our experimental results, we can think that an appropriate immune response or appropriate increase of cytokines will lead to cell proliferation and increase of cell viability. And excessive immune response or overexpression of some cytokines and genes will reduce cell proliferation and reduce cell activity. miR-217-5p regulates the expression of cytokines by inhibiting the expression of NOD1, thus affecting cell proliferation and cell viability. In summary, miR-217-5p plays an important regulatory role in inflammatory immune responses.

### NARL acts as a sponge for miR-217 to enhance NOD1 expression

**Figure 6:**
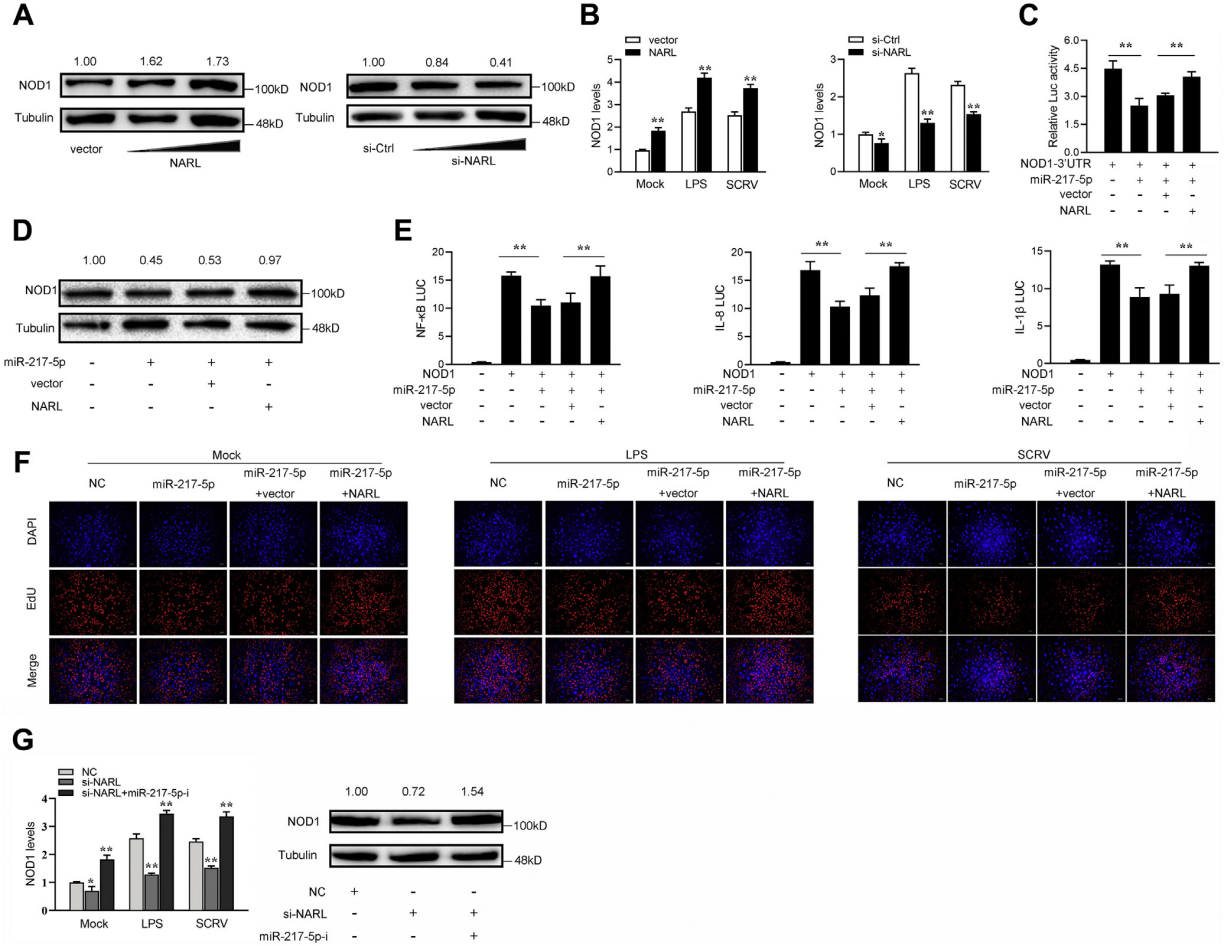
**NARL acts as a sponge for miR-217 to enhance NOD1 expression.***A* and *B*, relative mRNA and protein levels of NOD1 in MIC cells after cotransfected with si-NC or si-NARL and pcDNA3.1 vector or NARL expression plasmid by western blot assays (*A*) and qPCR (*B*). *C*, the relative luciferase activities were detected in EPC cells after cotransfection with NOD1 3’UTR luciferase reporter vector, NC, miR-217-5p, or NARL. *D*, western blot assays were detected in MIC cells after cotransfection with NOD1 overexpression plasmid, NC, miR-217-5p, or NARL. *E*, the relative luciferase activities were detected in MIC cells after cotransfection with NOD1 expression plasmid, phRL-TK Renilla luciferase plasmid, luciferase reporters, NC, miR-217-5p, or NARL. *F*, cell proliferation was assessed by EdU assays in MIC cells after cotransfected with NC, miR-217-5p, or NARL. *G*, relative mRNA and protein levels of NOD1 in MIC cells after cotransfected with NC, si-NARL, and si-NARL or miR-217-5p-i by qPCR (*left panel*) and western blot assays(*right panel*). All data represented the mean ± SD from three independent triplicated experiments. ∗*p* < 0.05; ∗∗*p* < 0.01.

Given that NARL interacts with miR-217-5p and miR-217-5p targets and regulates NOD1. Thus, we then tested whether NARL is able to regulate NOD1. NOD1 protein expression is significantly increased when NARL is overexpressed in MIC cells (left panel of Fig. 6*A*), while the NOD1 protein expression was significantly decreased through knockdown NARL (right panel of Fig. 6*A*). NARL expression plasmid or si-NARL is transfected into MIC cells and NOD1 expression levels were detected by qRT-PCR. The results show that NARL can indeed promote NOD1 expression while silencing NARL would significantly reduce NOD1 expression (Fig. 6*B*). These results indicated that miR-217-5p can inhibit NOD1 luciferase activity, while NARL can reverse the inhibition effect of miR-217-5p on NOD1 (Fig. 6*C*). Then, the MIC cells were cotransfected with miR-217-5p and NARL, and the western blotting assay showed that NARL could indeed reverse the inhibitory effect of miR-217-5p on NOD1 protein expression (Fig. 6*D*). Then, the NOD1 plasmid with full-length 3’UTR, miR-217-5p, NARL plasmid, and various reporter gene plasmids were cotransfected into EPC cells. The results showed that NARL could reverse the negative effect of miR-217-5p on the luciferase activities of NF-κB, IL-8, and IL-1β report genes (Fig. 6*E*). Moreover, we attempted to explore the effect of the NARL/miR-217-5p regulatory loop on cell proliferation. The results indicated that overexpression of NARL could counteract the negative effect of miR-217-5p on cell proliferation upon LPS or SCRV stimulation (Fig. 6*F*). We cotransfected miR-217-5p-i and si-NARL into MIC cells to further prove the results that NARL regulates NOD1 by modulating miR-217-5p. And the results show that miR-217-5p-i can reverse the negative effect of si-NARL on the NOD1 (Fig. 6*G*). Collectively, these data demonstrated that NARL serves as a ceRNA for miR-217-5p to regulate NOD1 expression.

### The ceRNA network of regulating NOD1 is widely found in teleost fish

**Figure 7:**
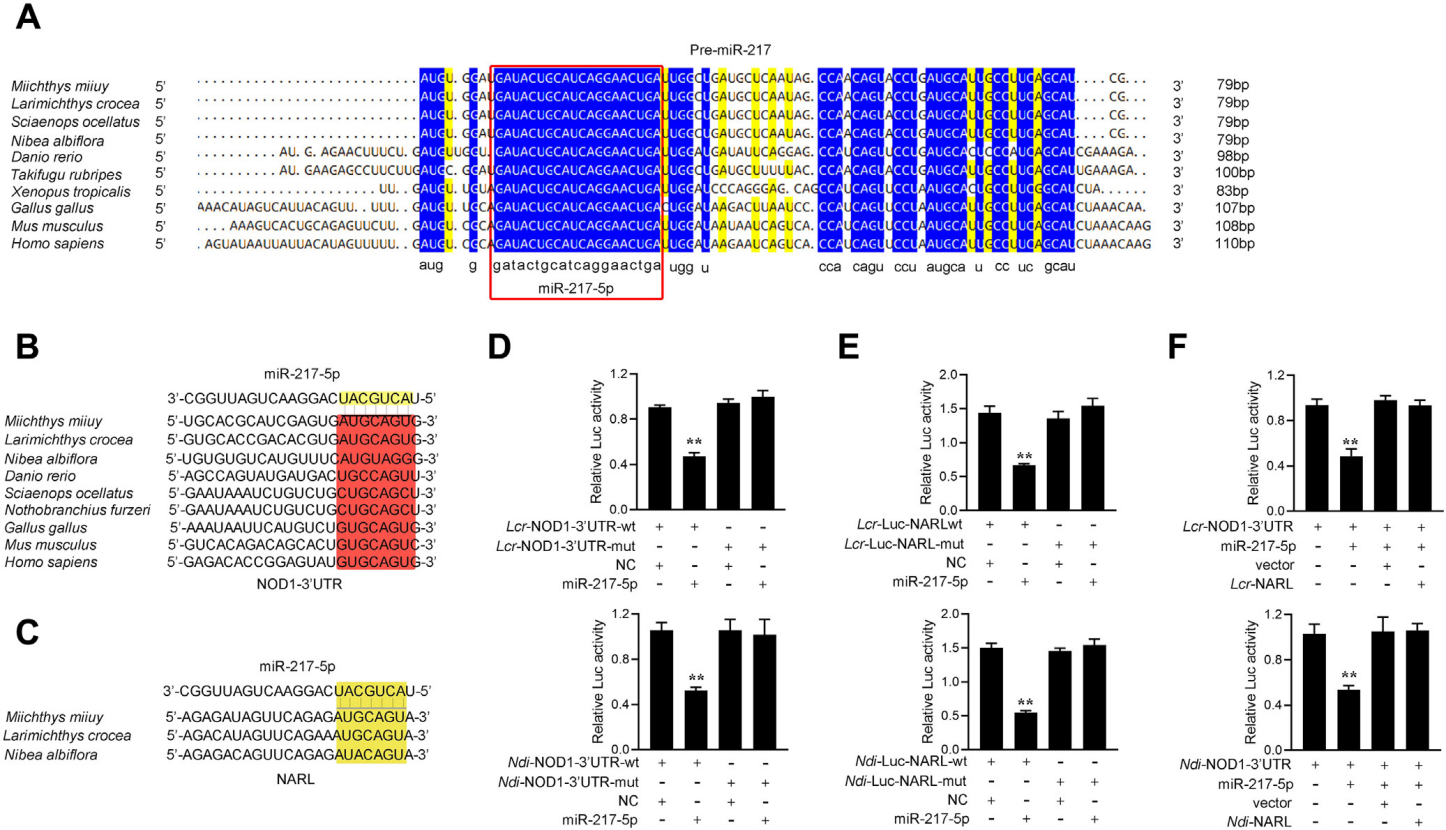
**The ceRNA network of regulating NOD1 is widely found in teleost fish.***A*, sequence alignment of pre-miR-217 from various vertebrate species. Mature miR-217 sequences are shown in boxes. *B*, putative miR-217-5p-binding site of NOD1 3’UTR among different vertebrate species. *C*, putative miR-217-5p-binding site of NARL among different vertebrate species. *D*, the relative luciferase activities were detected in EPC cells after cotransfected with *Lcr*-NARL-wt or *Lcr*-NARL-mut and miR-217-5p or NC (*upper panel*), and cells after cotransfected with *Ndi*-NARL-wt or *Ndi*-NARL-mut and miR-217-5p or NC (*lower panel*). *E*, the relative luciferase activities were detected in EPC cells after cotransfected with *Lcr*-NOD1-wt or *Lcr*-NOD1-mut and miR-217-5p or NC (*upper panel*) and in cells after cotransfected with *Ndi*-NOD1-wt or *Ndi*-NOD1-mut and miR-217-5p or NC (*lower panel*). *F*, the relative luciferase activities were detected in EPC cells after cotransfection with *Lcr*-NOD1- 3’UTR luciferase reporter vector, NC, miR-217-5p, or *Lcr*-NARL (*upper panel*), and EPC cells after cotransfection with *Ndi*-NOD1-3’UTR luciferase reporter vector, NC, miR-217-5p, or *Ndi*-NARL (*lower panel*). All data represented the mean ± SD from three independent triplicated experiments. ∗*p* < 0.05; ∗∗*p* < 0.01.

To address the generality of our findings, we first examined the sequence alignment of pre-miR-217 from different vertebrate species. Interestingly, as shown in Figure 7*A*, mature miR-217-5p displayed high conservation from mammals to fish. Further, the miR-217-5p binding site in NOD1 3’UTR also displayed high conservation from mammals to fish (Fig. 7*B*). To obtain the direct evidence that miR-217-5p could target NOD1 3’UTR across fish species, luciferase report plasmids were generated by cloning NOD1-3’UTR of *L. crocea* and *N. diacanthus* into pmirGLO vector, within the mutant devoid of miR-217-5p binding site as a control (Fig. 7*C*). Strikingly, miR-217-5p mimics were sufficient to decrease luciferase activities when respectively cotransfected with the wild types of *L. crocea* and *N. diacanthus* NOD1-3’UTR, whereas it shows no effect on the luciferase activity of cells transfected with their mutant-types (Fig. 7*D*). These results indicate that miR-217-5p can target the NOD1 gene in other fish species, which verifies that miR-217-5p is highly conserved among different species, and its function is also conserved to some extent.

Additionally, we also verified the findings that miR-217-5p regulating lncRNA NARL also exists in other species. To this end, we firstly examined the sequence alignment of NARL among different species. Interestingly, the sequence of NARL is highly conserved among different fish species. Particularly, NARL in these different species presents highly conserved in the binding sites of miR-217-5p (Fig. 7*B*). Then, we produced luciferase constructs of *L. crocea* and *N. diacanthus* NARL and their mutated forms with miR-217-5p binding sites mutated to investigate whether NARL in other fish species could also interact with miR-217-5p,. Luciferase assays revealed that miR-217-5p can suppress the luciferase activity of the wild-type of NARL luciferase plasmid in both species, but it has no effect on mutated forms (Fig. 7*E*). Furthermore, to test whether *L. crocea* and *N. diacanthus* NARL can affect miR-217-5p activity, we conducted luciferase assays and found that both *L. crocea* and *N. diacanthus* NARL could counteract the inhibitory effect of miR-217-5p on NOD1 (Fig. 7*F*). These results indicate that NARL may act as endogenous sponge RNA to interact with miR-217-5p among different fish. All of these data suggest that NARL contains relatively conserved elements among different fish species, which is very important for preserving their functions.

## Discussion

Both the host defense against pathogen invasion and the maintenance of the host's immune balance need to induce an appropriate immune response. Once the host recognizes the invasion of bacteria or viruses, it triggers a series of signal cascades that mediate the activation of NF-κB and IRF3, leading to the production of inflammatory cytokines and antiviral factors such as IFNs and triggering innate immune responses. The eradication of pathogenic infections requires timely and appropriate immune and inflammatory responses. However, excessive induction of inflammatory cytokines can lead to acute or chronic inflammatory diseases. Therefore, different levels of regulators and mechanism networks are needed to ensure the homeostasis of the immune system. In host cells, genes usually do not work alone, and they are usually combined into a “network” based on interaction. Herein, we discovered an interaction network regulating teleost NOD1-mediated antibacterial and antiviral signaling pathways. miR-217-5p can reduce the expression of NOD1 and suppress NOD1-mediated innate immune responses, which may be beneficial for bacterial or viral infection (18). Furthermore, in the present study, our results show that NARL acts as an endogenous sponge RNA to interact with miR-217-5p and enhances the inflammatory responses and antiviral responses. In short, NARL can counteract an increasing effect of miR-217-5p on LPS or SCRV stimulation, thus maintaining the stability of antibacterial and antiviral immune and ensuring appropriate immune responses. The data suggested the critical roles of long noncoding RNAs in modulating innate immune responses caused by bacterial or RNA viral infection and enriched that ceRNA regulation mechanism exists in teleost fish.

NLRs play a crucial role in recognizing bacterial or viral invasion, and NOD1, as one of the most concerned members of NLRs, is widely distributed in many tissues of organisms (32). As an intracellular receptor, NOD1 can effectively detect the pathogenic components produced by various Gram-negative bacteria in mammals (2). Recently, increasing studies have shown that NOD1 in teleost fish can activate the NF-κB signaling pathway and promote the host to produce various inflammatory cytokines to resist bacterial invasion (9, 10). In addition, NOD1 in fish can also act as the receptor of the RNA virus to enhance the immune response in the virus infection (11). In recent years, a series of regulatory factors have been shown to participate in the regulation of NOD1-mediated signal pathways in mammals, including coding genes (12, 13, 14, 15) and noncoding genes (16). Unlike mammals, the understanding of NOD1 and its signal pathway in teleost fish has just begun. Therefore, the research on the regulation of fish NOD1 signaling pathway is still very limited. And we previously reported for the first time that miR-144 and miR-217-5p can inhibit the expression of NOD1 at the posttranscriptional level, thereby inhibiting the inflammatory response mediated by NOD1 and reducing the immune strength in fish (17). In the present study, we found that the miR-217-5p/NOD1 axis can be further regulated by lncRNAs to achieve a more precise immune balance. Here, we identified that NARL acts as a ceRNA to participate in miR-217-5p/NOD1-mediated innate immune responses in fish.

**Figure 8:**
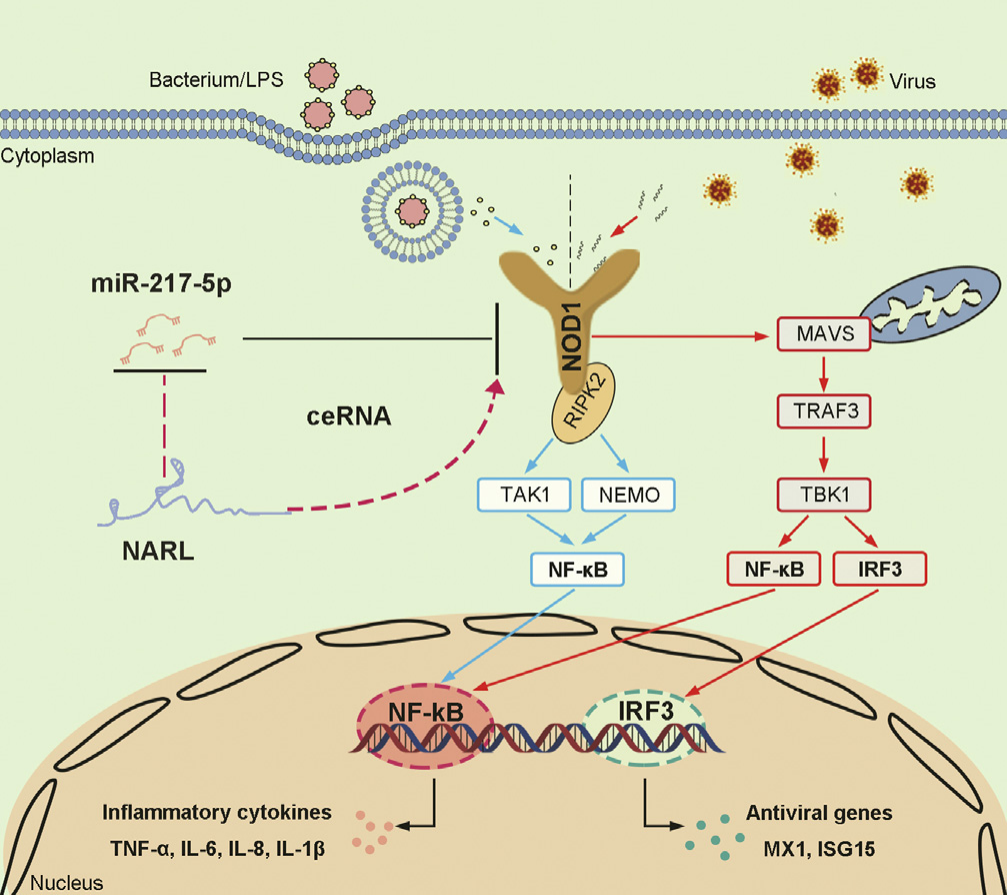
**The mechanism graph of the regulatory network and function of NOD1.** Fish NOD1 could induce the innate responses through recruiting NF-κB and IRF3 to trigger inflammatory cytokines or ISGs genes activation upon LPS or SCRV treatment. miR-217-5p targets NOD1 and represses NOD1-mediated antibacterial and antiviral responses, thereby helping the bacteria or virus escape the host's immune reaction. NARL acts as a molecular sponge regulating miR-217-5p to enhance NOD1 expression, thereby maintaining the stability of innate immune responses and ensuring appropriate inflammatory and antiviral responses. The ceRNA regulatory networks may exist wildly in vertebrate species. LPS, lipopolysaccharides.

The genomes of eukaryotes encode a large number of noncoding transcripts. It is reported that about two-thirds of mammalian transcripts are noncoding (33). We refer to these RNAs that do not encode proteins as ncRNAs, among them, miRNA, lncRNA, and circRNA have been extensively studied in mammals. Unlike most coding genes that can produce proteins, these ncRNAs genes act by controlling much larger messenger RNAs, which carry the protein-making instructions of the DNA. Increasing evidence showed that lncRNAs are not meaningless posttranscriptional by-products or genomic noise, but a class of ncRNAs that have strong regulatory effects on the coding genes in mammals. It is generally believed that lncRNA usually lacks strong conservation, such as the well-known lncRNA Xist, which has poor conservation (34). But it is worth noting that lncRNA also can evolve characteristics with certain conservative functions under moderate negative selection pressure (35). In the present study, we examined the sequence of NARL and found that it is conserved among some fish species, including miiuy croaker, large yellow croaker, and redfish (Fig. 8). Cross-species gene comparative analysis can be a powerful tool to study its functional evolution. Therefore, the conservation of lncRNA NARL sequences indicates its conservation of functions in fish, which has also been verified by experimental data. Although the conservation of some lncRNA is usually low, many lncRNAs still contain highly conserved elements, such as the binding site with miRNA (36). Here, we show an lncRNA NARL which can modulate NOD1 expression through competitively sponging miR-217-5p in miiuy croaker. Further, the miR-217-5p targeting sites at NARL have been shown conserved among several teleost fish, indicating that NARL may promote a similar biological function in different fish species.

Our previous study reveals that fish NOD1 plays an essential role in the innate antibacterial response (9, 10), then the researchers discovered fish NOD1 that promotes antiviral signaling by binding viral RNA and regulating the interaction of MDA5 and MAVS (11). In this study, we find that miR-217-5p plays a negative role, while NARL exhibits a positive regulatory role in regulating both inflammatory responses and antiviral responses. Furthermore, we identified that NARL acts as a regulator of inflammatory responses and antiviral responses *via* acting as a ceRNA for miR-217-5p to relieve its repressive effects on NOD1 expression; thus, immune homeostasis and immune balance are maintained. Our findings suggest the critical roles of lncRNAs in operating fish innate immune response processes, which will benefit for understanding the vertebrate immunology and the evolution of immune systems among vertebrates.

## Experimental procedures

### Sample and challenge

Miiuy croaker (∼50 g) was obtained from Zhoushan Fisheries Research Institute, Zhejiang Province, China. Fish was acclimated in aerated seawater tanks at 25 °C for 6 weeks before experiments. The challenge was performed as described previously (28, 37). Briefly, fish was challenged with 0.2 ml suspension of LPS (1 mg/ml) or treated with 0.2 ml SCRV through intraperitoneal. As comparison, 0.2 ml of physiological saline was used to challenge the individuals. Afterward, fishes were respectively sacrificed at different time points and the spleen samples were collected for RNA extraction. All animal experimental procedures were performed in accordance with the National Institutes of Health’s Guide for the Care and Use of Laboratory Animals, and the experimental protocols were approved by the Research Ethics Committee of Shanghai Ocean University (No. SHOU-DW-2018-047).

### Sequencing analysis and lncRNAs identification

The spleen tissues from three healthy fish, three SCRV challenged fish, and three LPS challenged fish were separated, and the total RNAs for the construction of the cDNA library were extracted. The cDNA libraries were sequenced using Illumina HiSeq 2500 platform. The RNA sequencing reads were aligned with the miiuy croaker genome using TopHat software (38), All transcripts longer than 200 bp were subjected to protein-coding potential evaluation by Coding Potential Calculator software, all noncoding transcripts were aligned to the Rfam database in order to eliminate all noncoding transcripts that had been previously annotated as rRNA, miRNA, or other small noncoding RNA transcripts (39). All remaining transcripts were identified as lncRNAs.

### Cell culture and treatment

*M. miiuy* intestine cells (MIC), *M. miiuy* kidney cells (MKC), and *M. miiuy* brain cells (MBrC), were cultured in L-15 medium (HyClone) supplemented with 10% fetal bovine serum (FBS; Gibco), 100 U/ml penicillin, and 100 μg/ml streptomycin at 26 °C. *Epithelioma papulosum cyprini* (EPC) cells were maintained in medium 199 (Invitrogen) supplemented with 10% FBS, 100 U/ml penicillin, and 100 mg/ml streptomycin at 26 °C in 5% CO2. For stimulation experiments, MIC cells were challenged with LPS concentration of 10 μg/ml and harvested at different times for RNA extraction.

### Plasmid construction and cell transfection

NOD1 expression plasmid of *M. miiuy*, NOD1 3’UTR reporter plasmid of *M. miiuy*, *Larimichthys crocea*, and *Nibea diacanthus* were constructed as described (17). To construct NARL luciferase genes, the sequences of NARL in *M. miiuy*, *N. diacanthus*, and *L. crocea* were cloned into pmirGLO luciferase reporter vector, respectively. The mutated forms with point mutations in the miR-217-5p binding site were synthesized using Mut Express II Fast Mutagenesis Kit V2 with specific primers (Table S1). Meanwhile, the sequences of NARL were inserted into the mVenus-C1 (Invitrogen), which included the sequence of enhanced GFP. Also, NARL expression plasmids were constructed by cloning the NARL sequence region of *M. miiuy*, *N. diacanthus*, and *L. crocea* into pcDNA3.1 vector. To build pcDNA3.1-MS2, the MS2-12X fragment was inserted into the *BamH* I and *EcoR* V restriction sites of pcDNA3.1 vector, and then the NARL was amplified and cloned into pcDNA3.1-MS2. The mutated forms with point mutations in the miR-217-5p binding site were synthesized using Mut Express II Fast Mutagenesis Kit V2 with specific primers (Table S1). A miR-217-5p sensor was created by inserting two consecutive miR-217-5p complementary sequences into the psiCHECK-2 vector (Promega). The correct construction of the plasmids was verified by Sanger sequencing and extracted through EndoFree Plasmid DNA Miniprep Kit (Tiangen). Transfections were performed using the Lipofectamine RNAiMAX and Lipofectamine 3000 (Invitrogen) according to the manufacturer’s instructions, respectively.

### RNA oligoribonucleotides

The miR-217-5p mimics are synthetic double-stranded RNAs (dsRNAs) with stimulating naturally occurring mature miRNAs. The miR-217-5p mimics sequences were 5′-UACUGCAUCAGGAACUGAUUGGC-3′ (sense) and 5′-CAAUCAGUUCCUGAUGCAGUAUU-3′ (antisense). The negative control RNA sequences were 5′-UUCUCCGAACGUGUCACGUTT-3′ (sense) and 5′-ACGUGACACGUUCGGAGAATT-3′ (antisense). miRNA inhibitors are synthetic single-stranded RNAs (ssRNAs) that sequester intracellular miRNAs and block their activity in the RNA interfering pathway. The miR-217-5p inhibitors sequence was 5′-GCCAAUCAGUUCCUGAUGCAGUA-3′. The negative control inhibitor sequence was 5′-CAGUACUUUUGUGUAGUACAA-3′. The RNA interference for NARL is as follows: si-NARL-1 sequence was 5′-GGCUAAAACCGCGAUGUAATT-3′; si-NARL-2 sequence was 5′-GAGUAUCGUCUUGUUUCAATT-3′. The negative control RNA sequences was 5′-UUCUCCGAACGUGUCACGUTT-3′ (sense) and 5′-ACGUGACACGUUCGGAGAATT-3′ (antisense).

### RNA extraction and quantitative real-time PCR

Both nuclear RNA and cytoplasmic RNA were extracted from MIC cells using the Cytoplasmic & Nuclear RNA Purification Kit (Norgen Biotek) according to the manufacturer’s instructions. Total RNA was isolated using TRIzol Reagent (Invitrogen), and cDNA was obtained by reverse transcriptional RNA using FastQuant RT Kit (Tiangen), which includes DNase treatment of RNA to eliminate genomic contamination. The nuclear and cytosolic fractions were separated using the Cytoplasmic & Nuclear RNA Purification Kit (Norgen Biotek) according to the manufacturer’s instructions. Gene expression was measured by using SYBR Premix Ex TaqTM (Takara). The small RNA was extracted by using miRcute miRNA Isolation Kit (Tiangen), and miRcute miRNA FirstStrand cDNA Synthesis Kit (Tiangen) was applied to reverse transcription of miRNAs. The expression analysis of miR-217-5p was executed by using the miRcute miRNA qPCR Detection Kit (Tiangen). Quantitative real-time PCR was performed in an Applied Biosystems QuantStudio 3 (Thermo Fisher Scientific). GAPDH and 5.8S rRNA were used as negative controls to detect mRNA, lncRNA, and miRNA expression, respectively. Primer sequences are displayed in Table S1.

### Dual-luciferase reporter assays

The NARL or NOD1-3’UTR wild-type and the mutant devoid of the miR-217-5p binding site were cotransfected with miR-217-5p mimics into EPC cells. At 24 h posttransfection, reporter luciferase activities were measured using the Dual-Luciferase reporter assay system (Promega). To determine the functional regulation of NARL or NOD1, MIC cells were cotransfected NOD1 expression plasmid or NARL expression plasmid, together with NF-κB, IL-8, and IL-1β luciferase reporter gene plasmids (17), phRL-TK Renilla luciferase plasmid, either miR-217-5p mimics or negative controls. At 48 h posttransfection, the cells were lysed for reporter activity using the Dual-Luciferase reporter assay system (Promega). The miR-217-5p sensor was cotransfected with miR-217-5p mimics or NARL expression plasmid into MIC cells. At 48 h posttransfection, the cells were lysed for reporter activity. All the luciferase activity values were achieved against the renilla luciferase control. The transfection of each construct was performed in triplicate in each assay. Ratios of renilla luciferase readings to firefly luciferase readings were taken for each experiment, and triplicates were averaged.

### Western blotting

Cells were lysed and collected in 1×sodium salt-solyacrylamide gel electrophoresis loading buffer. The protein content in the collected cell lysates was measured with the BCA protein detection kit (Vazyme), then subjected to sodium salt-solyacrylamide gel electrophoresis (10%) gel, and transferred to polyvinylidene difluoride (Millipore) membranes by semidry blotting (Bio-Rad Trans Blot Turbo System). The membrane is sealed with 5% bovine serum albumin. Protein was with different antibodies. The antibody against NOD1 was diluted at 1: 1000 (Genscript); anti-Flag, anti-GFP, and anti-Tubulin monoclonal antibody were diluted at 1: 2000 (Beyotime); HRP-conjugated anti-rabbit IgG or anti-mouse IgG (Abbkine) at 1: 5000. The results were representative of three independent experiments. The immunoreactive proteins were detected by using WesternBright ECL (Advansta). The digital imaging was performed with a cold CCD camera.

### RNA pull-down assay

NARL and NARL-mut with miR-217-5p binding sites mutated were transcribed *in vitro*. The two transcripts were biotin-labeled with the T7 RNA polymerase and Biotin RNA Labeling Mix (Roche), treated with RNase-free DNase I and purified with an RNeasy Mini Kit (Qiagen). The whole-cell lysates from MIC cells (∼1.0 × 10^7^) were incubated with purified biotinylated transcripts for 1 h at 25 °C. The complexes were isolated by streptavidin agarose beads (Invitrogen). RNA was extracted from the remaining beads, and qPCR was used to evaluate the expression levels of miRNAs. The specific protocol of pull-down assay as described (40, 41).

### RNA immunoprecipitation (RIP) assay

To explore whether NARL is involved in the ceRNA regulation process, MIC cells (∼2.0 × 10^7^) were cotransfection with pcDNA3.1-flag, pcDNA3.1-AGO2-flag, or miR-217-5p for RNA immunoprecipitation (RIP) assays. To prove that NARL has the ability to directly bind to miR-217, MIC cells (∼2.0 × 10^7^) were cotransfection with pcDNA3.1-MS2, pcDNA3.1-MS2-NARL, pcDNA3.1-MS2-NARL-mut, pcDNA3.1-MS2-NOD1-3’UTR, or pMS2-GFP for RIP assays. In short, MIC cells were harvested after 48 h transfection, and RIP assays were carried out with Magna RIP RNA-Binding Protein Immunoprecipitation Kit (Millipore) and anti-GFP antibody (Abcam) following the manufacturer’s protocol. RNA was extracted from the beads, and the expression level of miRNA was evaluated by qRT-PCR (40).

### Cell viability and proliferation

Cell viability was measured 48 h after transfection in LPS-treated MIC with Celltiter-Glo Luminescent Cell Viability assays (Promega) according to the manufacturer’s instructions (10). At the same time, cell proliferation was measured with BeyoClick EdU cell Proliferation Kit following the manufacturer’s instructions (Beyotime). All the experiments were performed in triplicate.

### Flow cytometric analysis of apoptosis

The apoptotic assay was conducted using an Annexin V-FITC Apoptosis Detection Kit (Beyotime) and analyzed with flow cytometry (Beckman). The ratio of early and late apoptotic cells was detected to calculate the apoptotic rate (42).

### Statistical analysis

Data are expressed as the mean ± SD from at least three independent triplicated experiments. Student’s *t*-test was used to evaluate the data. The relative gene expression data was acquired using the 2 ^ΔΔCT^ method, and comparisons between groups were analyzed by one-way analysis of variance (ANOVA) followed by Duncan’s multiple comparison tests (43). A value of *p* < 0.05 was considered significant.

## Data availability

RNA sequencing data have been deposited in the Sequence Read Archive (SRA) at the National Center for Biotechnology Information (NCBI) under accession number PRJNA685924 and PRJNA691457. All other data are contained within the article.

## Supporting information

This article contains supporting information.

## Conflict of interests

The authors declare that they have no conflicts of interest with the contents of this article.
