# Supplementary material for: The long noncoding RNA NARL regulates immune responses via microRNA-mediated NOD1 downregulation in teleost fish
Source: J Biol Chem. 2021 Feb 11;296:100414. doi: 10.1016/j.jbc.2021.100414 (PMC7966872; doi:10.1016/j.jbc.2021.100414)
Supplement: Supplemental Table S1 [file mmc1.pdf]

Supplemental Table 1. PCR primer information in this study.

**Primer**	**Sequence (5’-3’)**
**Real-time PCR**	
NOD1-RT-F	TCGCACTCGTATTGGATG
NOD1-RT-R	CACTGGTGGAAAGGTAGG
NARL-RT-F	GAGACAGTTCAACGAGGAC
NARL-RT-R	TGCGGATGATGACAGAT
TNF-α-RT-F	GTTTGCTTGGTACTGGAATGG
TNF-α-RT-R	TGTGGGATGATGATCTGGTTG
IL-1β-RT-F	CATAAGGATGGGGACAACGAG
IL-1β-RT-R	TAGGGGACGGACACAAGGGTA
IL-6-RT-F	GCGGTAAAGGCATGGATAT
IL-6-RT-R	GTTGTAGTTGGAAGGGCAG
IL-8-RT-F	AGCAGCAGAGTCTTCGT
IL-8-RT-R	TCTTCGCAGTGGGAGTT
GAPDH-RT-F	ACCTTCACTCCTCCATCTT
GAPDH-RT-R	AGGTCACAGACACGGTTG
miR-217-RT-F	AGTACTGCATCAGGAACTGA
miR-217-RT-R	CCAGTTTTTTTTTTTTTTTGCCAATC
5.8S-RT-F	AACTCTTAGCGGTGGATCA
5.8S-RT-R	GTTTTTTTTTTTTTTTGCCGAGTG
**Vector construction**	
NOD1-pcDNA3.1-F	GACGATGACGACAAGAAGCTTATGTCGTGCCTGAACATCCTCA
NOD1-pcDNA3.1-R	TTAGTGCCAGTCAGTGGAAGCGCAGCCTC
NOD1-3’UTR-F	CGCGAGCTCAACAGAAACAGTTTGAGG
NOD1-3’UTR-R	TGCTCTAGACTTCTTTCATTCACATTG
NOD1-3’UTR-mut-F	GAGTGTAAGTCAGTGTCTGCTTCAATGGGAATACTG
NOD1-3’UTR-mut-R	GACACTGACTTACACTCGATGCGTGCACGTTC
NOD1-3’UTR-mVenus-F	CCCAAGCTTCGCTTCCACTGACTGGCACT
NOD1-3’UTR-mVenus-R	CGCGGATCCCACCAGCTATGAGCACCAGG
NOD1-3’UTR-mVenus-mut-F	GAGTGTAAGTCAGTGTCTGCTTCAATGGGAATACTG
NOD1-3’UTR-mVenus-mut-R	GACACTGACTTACACTCGATGCGTGCACGTTC
pcDNA3.1-MS2-NOD1-3’UTR-F	ACTATAGGGAGACCCAAGCTTATGTAAAAATATGATGACATATGGTTAGAAT
pcDNA3.1-MS2-NOD1-3’UTR-R	GCGGCCGTTACTAGTGGATCCAAACAAGCATACACACGAGTTATACAGC
NARL-pcDNA3.1-F	ACTATAGGGAGACCCAAGCTTAGCTGCCCGCGCACAACT
NARL-pcDNA3.1-R	TGATGGATATCTGCAGAATTCCTATGCAAACTTTTACGCATGACTC
NARL-pmirGLO-F	TGTTTAAACGAGCTCGCTAGCGGAAGAGGGCGTAGGCTCG
NARL-pmirGLO-R	CAGGTCGACTCTAGACTCGAGGTGATTCTTTCCTACACACGGTAGAA
NARL-pmirGLO-mut-F	CAGAACCTACTGCGTTTCTTTTTCTGAGACTAATAAATGCA
NARL-pmirGLO-mut-R	AAACGCAGTAGGTTCTGAACTATCTCTTTAACCAAATCACA
NARL-mVenus-F	TCAGATCTCGAGCTCAAGCTTGGAAGAGGGCGTAGGCTCG
NARL-mVenus-R	CGGGCCCGCGGTACCGTCGACGTGATTCTTTCCTACACACGGTAGAA

NARL-mVenus-mut-F	CAGAACCTACTGCGTTTCTTTTTCTGAGACTAATAAATGCA
NARL-mVenus-mut-R	AAACGCAGTAGGTTCTGAACTATCTCTTTAACCAAATCACA
pcDNA3.1-MS2-NARL-F	ACTATAGGGAGACCCAAGCTTAGCTGCCCGCGCACAACT
pcDNA3.1-MS2-NARL-R	GCGGCCGTTACTAGTGGATCCCTATGCAAACTTTTACGCATGACTC
pcDNA3.1-MS2-NARL-mut-F	CAGAACCTACTGCGTTTCTTTTTCTGAGACTAATAAATGCA
pcDNA3.1-MS2-NARL-mut-R	AAACGCAGTAGGTTCTGAACTATCTCTTTAACCAAATCACA
NARL-T7-F	TAATACGACTCACTATAGGGAGCTGCCCGCGCACAACT
NARL-T7-R	CTATGCAAACTTTTACGCATGACTC
*Lcr*NOD1-3'UTR-F	CGCGAGCTCACTGGCACTAACGAGAGG
*Lcr*NOD1-3'UTR-R	TGCTCTAGAAAGGTCCATGAAGGAGGA
*Lcr*NOD1-3'UTR-mut-F	ACGTGTAAGTCAGTGTGTGTTCACACTATCCGCTTC
*Lcr*NOD1-3'UTR-mut-R	CACACTGACTTACACGTGTCGGTGCACGTTCC
*Lcr*NARL-pcDNA3.1-F	ACTATAGGGAGACCCAAGCTTAGCTGCCCGCGCACAACT
*Lcr*NARL-pcDNA3.1-R	TGATGGATATCTGCAGAATTCCTATGCAAACTTTTACGCATGACTC
*Lcr*NARL-pmirGLO-F	TGTTTAAACGAGCTCGCTAGCGGAAGAGGGCGTAGGCTCG
*Lcr*NARL-pmirGLO-R	CAGGTCGACTCTAGACTCGAGGTGATTCTTTCCTACACACGGTAGAA
*Lcr*NARL-pmirGLO-mut-F	CAGAACCTACTGCGTTTCTTTTTCTGAGACTAATAAATGCA
*Lcr*NARL-pmirGLO-mut-R	AAACGCAGTAGGTTCTGAACTATCTCTTTAACCAAATCACA
*Ndi*NOD1-3'UTR-F	CGCGAGCTCGAAACCAGCTCACTGACC
*Ndi*NOD1-3'UTR-R	TGCTCTAGACACATTGAATGCACCACA
*Ndi*NOD1-3'UTR-mut-F	GAGTGTAAGTCAGTGTGTGTTCACACTGTCCGCT
*Ndi*NOD1-3'UTR-mut-R	CACACTGACTTACACTCGATCGTGCCGTTCCT
*Ndi*NARL-pcDNA3.1-F	ACTATAGGGAGACCCAAGCTTAGCTGCCCGCGCACAACT
*Ndi*NARL-pcDNA3.1-R	TGATGGATATCTGCAGAATTCCTATGCAAACTTTTACGCATGACTC
*Ndi*NARL-pmirGLO-F	TGTTTAAACGAGCTCGCTAGCGGAAGAGGGCGTAGGCTCG
*Ndi*NARL-pmirGLO-R	CAGGTCGACTCTAGACTCGAGGTGATTCTTTCCTACACACGGTAGAA
*Ndi*NARL-pmirGLO-mut-F	CAGAACCTACTGCGTTTCTTTTTCTGAGACTAATAAATGCA
*Ndi*NARL-pmirGLO-mut-R	AAACGCAGTAGGTTCTGAACTATCTCTTTAACCAAATCACA
miR-217-5p-sensor-F	TCGAGTTGTCATTCTCAAATACACGACTTGTCATTCTCAAATACACGACGC
miR-217-5p sensor-R	GGCCGCGTCGTGTATTTGAGAATGACAAGTCGTGTATTTGAGAATGACAAC
