# Supplementary material for: The long noncoding RNA NARL regulates immune responses via microRNA-mediated NOD1 downregulation in teleost fish
Source: J Biol Chem. 2021 Feb 11;296:100414. doi: 10.1016/j.jbc.2021.100414 (PMC7966872; doi:10.1016/j.jbc.2021.100414)
Supplement: Supplemental Table S2 [file mmc2.pdf]

Supplemental Table 2. The differentially expressed lncRNAs obtained after SCRV and LPS stimulation.

**lncRNA**	**SCRV-Ctrl**			**LPS-Ctrl**		
	**log2Fold Change**	***p*-value**	**regulation**	**log2Fold Change**	***p*-value**	**regulation**
MSTRG.10039	-2.84	0.15	down	-2.27	0.26	down
MSTRG.10537	-1.93	0.29	down	-1.87	0.32	down
MSTRG.10671	-1.65	0.23	down	-1.62	0.24	down
MSTRG.10696	1.07	0.40	up	1.61	0.36	up
MSTRG.108	-1.27	0.67	down	1.64	0.27	up
MSTRG.10943	1.19	0.25	up	-2.19	0.16	down
MSTRG.10948	-1.09	0.49	down	-1.86	0.36	down
MSTRG.11011	-6.36	0.02	down	-1.13	0.65	down
MSTRG.11012	1.26	0.63	up	1.82	0.48	up
MSTRG.11093	-1.39	0.23	down	2.46	0.33	up
MSTRG.1119	1.38	0.57	up	1.76	0.37	up
MSTRG.11427	-1.35	0.26	down	-1.14	0.35	down
MSTRG.11555	1.14	0.30	up	1.22	0.28	up
MSTRG.11586	-9.68	0.00	down	-1.00	0.64	down
MSTRG.11605	-12.67	0.00	down	-1.67	0.06	down
NARL	14.05	0.00	up	14.14	0.00	up
MSTRG.11620	-1.17	0.33	down	1.48	0.27	up
MSTRG.11622	-1.23	0.30	down	1.34	0.21	up
MSTRG.11624	-1.05	0.47	down	-1.03	0.50	down
MSTRG.11677	1.98	0.17	up	-1.07	0.31	down
MSTRG.11749	2.25	0.08	up	2.77	0.21	up
MSTRG.1175	-1.25	0.30	down	-1.69	0.16	down
MSTRG.11898	-1.53	0.17	down	-1.77	0.14	down
MSTRG.12133	5.07	0.03	up	-5.41	0.00	down
MSTRG.12536	1.29	0.34	up	-2.26	0.05	down
MSTRG.12658	1.28	0.62	up	1.66	0.41	up
MSTRG.12686	-13.59	0.00	down	-1.45	0.57	down
MSTRG.12839	1.35	0.59	up	1.32	0.12	up
MSTRG.13069	-1.08	0.50	down	17.23	0.00	up
MSTRG.13102	1.88	0.45	up	-1.22	0.43	down
MSTRG.13159	-4.92	0.05	down	-13.01	0.00	down
MSTRG.13477	-1.83	0.17	down	-1.82	0.20	down
MSTRG.13828	-2.75	0.17	down	-1.09	0.53	down
MSTRG.13889	1.41	0.44	up	-1.17	0.59	down
MSTRG.13977	-4.14	0.01	down	1.27	0.34	up
MSTRG.14019	-2.04	0.16	down	-4.83	0.00	down
MSTRG.14034	-1.26	0.45	down	-1.49	0.27	down
MSTRG.14085	1.98	0.13	up	1.32	0.31	up

MSTRG.14095	-11.78	0.00	down	-1.83	0.36	down
MSTRG.14150	2.39	0.16	up	-2.31	0.10	down
MSTRG.14169	-1.87	0.17	down	-1.60	0.24	down
MSTRG.14260	-14.09	0.00	down	-14.09	0.00	down
MSTRG.14261	-3.52	0.17	down	-1.09	0.59	down
MSTRG.14373	-13.05	0.00	down	-4.21	0.03	down
MSTRG.14455	-3.44	0.09	down	15.52	0.00	up
MSTRG.14566	1.42	0.18	up	-1.70	0.14	down
MSTRG.14578	-4.98	0.03	down	-4.99	0.02	down
MSTRG.14790	-13.17	0.00	down	-13.17	0.00	down
MSTRG.14805	3.68	0.04	up	3.74	0.04	up
MSTRG.14807	-8.24	0.00	down	-1.06	0.68	down
MSTRG.14839	-2.34	0.26	down	-1.97	0.25	down
MSTRG.15193	-1.99	0.12	down	-1.01	0.39	down
MSTRG.15662	-2.57	0.22	down	1.42	0.21	up
MSTRG.15702	4.30	0.09	up	5.33	0.03	up
MSTRG.15705	1.32	0.30	up	1.30	0.30	up
MSTRG.15722	1.60	0.46	up	2.50	0.15	up
MSTRG.15732	-1.72	0.40	down	-12.27	0.00	down
MSTRG.15761	-1.57	0.55	down	-1.18	0.46	down
MSTRG.15821	-1.57	0.39	down	-1.43	0.37	down
MSTRG.15906	-1.18	0.36	down	1.05	0.40	up
MSTRG.16060	-2.67	0.10	down	-4.06	0.01	down
MSTRG.16127	-12.33	0.00	down	-1.19	0.65	down
MSTRG.16542	-1.49	0.48	down	3.95	0.12	up
MSTRG.16543	-1.76	0.06	down	-1.20	0.18	down
MSTRG.16549	3.27	0.04	up	2.48	0.13	up
MSTRG.16810	1.04	0.40	up	2.73	0.28	up
MSTRG.16915	-1.36	0.28	down	1.87	0.26	up
MSTRG.16949	-1.52	0.25	down	-1.55	0.37	down
MSTRG.16958	17.80	0.00	up	17.39	0.00	up
MSTRG.16986	1.21	0.48	up	1.24	0.47	up
MSTRG.16997	-1.45	0.22	down	-1.46	0.25	down
MSTRG.16998	-2.71	0.18	down	-2.03	0.12	down
MSTRG.17030	-5.35	0.01	down	-1.74	0.22	down
MSTRG.17055	13.64	0.00	up	-1.60	0.31	down
MSTRG.17093	-1.65	0.26	down	-1.94	0.16	down
MSTRG.17118	1.64	0.35	up	-1.98	0.54	down
MSTRG.17143	-3.35	0.16	down	-1.15	0.50	down
MSTRG.17200	1.34	0.25	up	1.18	0.35	up
MSTRG.17210	-1.51	0.28	down	1.87	0.19	up
MSTRG.17265	-2.45	0.14	down	-1.20	0.50	down
MSTRG.17380	1.13	0.39	up	1.03	0.44	up

MSTRG.17607	-3.44	0.00	down	-3.71	0.00	down
MSTRG.17608	3.15	0.00	up	2.76	0.01	up
MSTRG.17642	-1.09	0.32	down	-1.05	0.43	down
MSTRG.17710	-6.26	0.02	down	-4.57	0.08	down
MSTRG.17718	1.36	0.36	up	1.14	0.43	up
MSTRG.17816	-4.34	0.03	down	-1.04	0.49	down
MSTRG.1795	2.02	0.53	up	-10.74	0.00	down
MSTRG.17989	-12.26	0.00	down	1.29	0.23	up
MSTRG.18064	1.75	0.39	up	1.65	0.24	up
MSTRG.18115	2.25	0.36	up	3.06	0.22	up
MSTRG.18145	1.38	0.48	up	1.23	0.61	up
MSTRG.18201	-2.08	0.27	down	-1.53	0.43	down
MSTRG.18208	-1.19	0.27	down	-1.33	0.25	down
MSTRG.18240	-3.75	0.12	down	-6.80	0.01	down
MSTRG.18260	-1.13	0.41	down	-1.32	0.35	down
MSTRG.18261	12.61	0.00	up	-3.21	0.17	down
MSTRG.18296	-3.40	0.04	down	-4.11	0.05	down
MSTRG.18526	1.85	0.09	up	1.38	0.20	up
MSTRG.18572	1.04	0.45	up	1.75	0.20	up
MSTRG.18625	-2.13	0.25	down	-1.89	0.36	down
MSTRG.19035	-1.75	0.22	down	-1.85	0.20	down
MSTRG.19036	-1.80	0.46	down	-6.56	0.01	down
MSTRG.19088	-1.22	0.35	down	-1.21	0.36	down
MSTRG.19096	-12.06	0.00	down	-6.14	0.02	down
MSTRG.19148	-5.56	0.01	down	1.35	0.45	up
MSTRG.19200	-1.18	0.33	down	-1.53	0.28	down
MSTRG.19238	1.99	0.28	up	1.99	0.18	up
MSTRG.19249	5.95	0.00	up	-2.81	0.03	down
MSTRG.19326	13.16	0.00	up	13.16	0.00	up
MSTRG.19390	-2.50	0.02	down	-1.11	0.29	down
MSTRG.19527	-2.05	0.14	down	-1.58	0.22	down
MSTRG.19528	-2.51	0.11	down	-1.58	0.24	down
